# Research on Thermal Insulation and Durability of Bio-Based Thermal Insulation Materials and Its Prospect of Engineering Application

**DOI:** 10.3390/ma19061229

**Published:** 2026-03-20

**Authors:** Sen Luo, Shuo Wang, Chi Hu, Lirui Feng, Haihong Fan, Hongqiang Ma

**Affiliations:** 1 China Railway Tiegong City Construction Co., Ltd., Jinan 250000, China; 2 Engineering Research Center of Zero-Carbon Energy Buildings and Measurement Techniques, Ministry of Education, Hebei University, Baoding 071002, China

**Keywords:** bio-based thermal insulation materials, thermal insulation performance, thermal conductivity, durability, low carbon

## Abstract

This study takes the relevant literature published in the past decade as the research object, screens the literature by setting clear inclusion and exclusion criteria, and systematically reviews the thermal insulation performance, durability, and prospects for engineering applications of bio-based thermal insulation materials by means of qualitative integration and comparative analysis. With the advantages of low energy consumption, renewability, and biodegradability, bio-based thermal insulation materials have emerged as a green alternative to traditional thermal insulation materials. This paper systematically reviews the research progress of such materials, which are classified into two categories: natural biomass (e.g., straw bales and cork boards) and bio-based composites. The core thermal insulation indicators include thermal conductivity, thermal resistance, and thermal storage coefficient, and the performance is affected by factors such as component ratio, pore structure, temperature, and humidity. The thermal conductivity of some bio-based materials is comparable to that of expanded polystyrene (EPS) and mineral wool. In terms of durability, temperature–humidity cycling, corrosion, biological erosion, and mechanical action are the main causes of performance degradation, and composite modification can effectively improve their stability. Current engineering applications face challenges such as thermal insulation performance being susceptible to humidity, poor construction compatibility, high costs, and a lack of relevant standards. Future research should focus on the development of high-performance composite systems, the investigation of long-term durability mechanisms, the innovation of low-cost green preparation technologies, and the establishment of unified standards, so as to promote the large-scale application of bio-based thermal insulation materials in the construction industry and contribute to the achievement of carbon neutrality goals.

## 1. Introduction

As one of the industries with the highest energy consumption worldwide, the construction industry’s carbon emission reduction efficacy directly impacts the achievement of global sustainable development goals [1]. Embodied energy refers to the total energy consumed throughout the entire production process of materials; carbon footprint represents the total carbon emissions generated during the life cycle of materials, and carbon emission focuses on the amount of carbon released in specific stages. By 2021, carbon emissions from this industry had accounted for 37% of the global total, mainly stemming from the high energy consumption throughout the entire life cycle of building materials, including production, transportation, construction, and building operation [2]. Massive carbon emissions have exacerbated the greenhouse effect and climate change; thus, promoting energy conservation, emission reduction, and the transformation toward a sustainable development model in the construction industry has become a global consensus [3]. Enhancing the thermal insulation performance of building envelope structures is a core approach to reducing building operational energy consumption. Among these structures, external wall panels account for more than 80% of the building’s external envelope area, and the selection of their materials plays a pivotal role in controlling building energy consumption and reducing greenhouse gas emissions [4]. Although traditional thermal insulation materials such as mineral wool and expanded polystyrene (EPS) have been widely used in external wall insulation, they suffer from drawbacks, including high embodied energy, non-biodegradability, and heavy reliance on petrochemical resources, which conflict with the concept of low-carbon development [5].

Biomass materials derived from plant or animal biomass have achieved considerable development in many fields [6,7,8]. Benefiting from the mature technical applications in other sectors, these materials have paved a new path for the carbon-neutral development of the construction industry. The production energy consumption of biomass materials such as bamboo, wood, and agricultural residues is significantly lower than that of traditional building materials; moreover, they can sequester carbon dioxide from the atmosphere during their growth cycle. Based on the viewpoints of the existing literature [9,10,11], this characteristic can not only reduce carbon emissions during the building operation phase but also has the potential to achieve zero-carbon or even negative carbon emissions throughout the whole life cycle of buildings. Meanwhile, their renewability and biodegradability are aligned with China’s circular economy principles and sustainable development goals, which is of great significance for the construction industry to reach carbon peaking and carbon neutrality, and injects new impetus into the development of green buildings.

At present, a large number of studies have focused on the performance comparison between bio-based thermal insulation materials and traditional counterparts, with the core concentrated on two major dimensions: physical performance and environmental performance [4,12,13,14]. Liu et al. [15] defined bio-based thermal insulation materials as “composite thermal insulation materials composed solely of naturally sourced biological materials, including forest/agricultural residues, recycled materials, and fast-growing economic plants, or formed by mixing these materials with binders and additives in specific proportions”, and verified that such materials are often superior to traditional materials like mineral wool and expanded polystyrene (EPS) in terms of energy efficiency (EE) and environmental carbon footprint (EC) [12,16]. Typical bio-based materials such as hemp concrete, cork, and straw bales realize efficient thermal insulation by virtue of their natural pore structures, and their embodied energy is far lower than that of synthetic materials. Their application in buildings can significantly reduce the reliance on heating and cooling systems, thereby decreasing operational energy consumption and carbon emissions [17,18,19]. Due to space constraints, this paper does not conduct the calculation and system boundary definition of life cycle assessment (LCA), and all the conclusions drawn are derived from the literature citations mentioned above.

However, existing research still has obvious limitations. First, there are controversies regarding the assessment of environmental performance. Grazieschi et al. [5] found that bio-based thermal insulation materials have higher uncertainties in energy efficiency and carbon emissions than traditional materials. A study by Fuchsl et al. further showed that in more than one-third of cases, the energy efficiency and carbon emission performance of hemp-based materials were not superior to those of EPS [20]. A unified conclusion has not yet been reached on the environmental potential of such materials at the material level. Regarding the contradictory conclusions of the aforementioned LCA results and environmental performance studies, the discrepancies stem from three main reasons. First, there are differences in the setting of LCA system boundaries across different studies, with some failing to cover links such as raw material transportation and waste disposal. Second, the data sources vary, leading to deviations between laboratory simulation data and actual engineering monitoring data. Third, the selection of evaluation methods is subjective, as different models adopt inconsistent accounting standards for carbon sinks and energy consumption. Second, research scenarios are biased toward laboratory settings. Studies on carbon footprint, thermal insulation, sound absorption, and mechanical behavior are mostly based on controlled environments, lacking sufficient verification data from actual construction projects [15]. Under laboratory conditions, the durability tests of materials usually employ accelerated aging tests involving single or a limited number of influencing factors. However, in actual building applications, materials have to withstand the combined effects of multiple factors such as temperature–humidity cycling, corrosion, and biological erosion. This leads to a significant discrepancy between the service life derived from laboratory tests (usually up to 15–20 years) and the operational lifespan in practical engineering projects (generally 10–15 years). Third, performance evaluation is not comprehensive enough. Existing research mostly focuses on single performance indicators in isolation, and insufficient exploration has been conducted on the synergistic variation laws of thermal insulation performance and durability of materials under different environmental conditions such as temperature and humidity fluctuations, corrosion, and mechanical wear. This leads to diversity and inconsistency in material performance during practical applications, thus increasing the complexity of engineering implementation [10]. In addition, the expansion of bio-based materials in engineering applications is still concentrated in fields such as structural components and lightweight structures. Their large-scale application in thermal insulation systems still faces unresolved issues, including performance stability and construction compatibility [21]. Therefore, there is an urgent need for a comprehensive review that evaluates the thermal insulation performance and durability of bio-based thermal insulation materials, which can fill the knowledge gap between theoretical research and engineering applications and provide scientific support for their mainstream integration into the traditional architectural design process.

Based on a systematic review of the relevant literature in recent years, and aiming at the limitations of existing research, this study focuses on the following core objectives: first, to systematically integrate the core thermal insulation performance indicators (e.g., thermal conductivity, thermal resistance, and thermal storage coefficient) of bio-based thermal insulation materials and their influencing factors, including material composition, pore structure, and density; second, to comprehensively analyze the variation rules and degradation mechanisms of material durability under different environmental conditions, such as temperature–humidity cycling, chemical corrosion, biological erosion, and mechanical action; third, to clarify the controversial focuses of the environmental performance of bio-based thermal insulation materials and identify their advantages and bottlenecks in engineering applications; and fourth, to provide targeted references for the performance optimization, process improvement, and large-scale engineering application of bio-based thermal insulation materials. Compared with the existing review literature, the core innovation and unique contribution of this study lie in the following aspects: existing reviews mostly focus on a single dimension of either thermal insulation performance or durability in isolation, or only concentrate on the optimization of material preparation processes. In contrast, this study constructs a synergistic evaluation framework of “thermal insulation performance–durability” for the first time, while correlating key influencing factors such as moisture absorption sensitivity and mechanical properties, thus filling the research gap in multi-performance coupling studies. In addition, this study establishes a bridge between theoretical research and technological transformation by comparing laboratory data with actual engineering data, which is a content not covered by most existing reviews.

The scientific value and contribution of this study are mainly reflected in three aspects: First, from the perspective of synergistic analysis, it fills the research gap in the correlation between the thermal insulation performance and durability of bio-based thermal insulation materials, breaking the limitations of single-performance research. Second, it establishes a bridge linking laboratory test data with the service performance of actual engineering, providing a scientific basis for material technological transformation. Third, it objectively sorts out the controversial conclusions in this field, clarifies the core directions of future research, and offers a systematic reference framework for researchers in this field.

## 2. Research Methodology

To ensure the systematicity and rigor of this review, this study strictly follows the standardized procedure for systematic literature reviews, which specifically includes three stages: literature retrieval, screening, and analysis.

### 2.1. Literature Retrieval

Retrieval Databases: Four major databases, including Web of Science, China National Knowledge Infrastructure (CNKI), Elsevier, and SpringerLink, were selected, covering core research achievements in both Chinese and English.

Time Scope of Retrieval: 2015–2025, focusing on the latest research progress over the past decade.

Search Terms: The Chinese search terms included “bio-based thermal insulation materials”, “biomass insulation materials”, “thermal insulation performance”, “durability”, and “engineering application”; the English search terms included “bio-based thermal insulation materials”, “biomass insulation materials”, “thermal insulation performance”, “durability”, and “engineering application”. A combined subject term retrieval strategy was adopted to ensure the coverage of the relevant literature.

### 2.2. Inclusion and Exclusion Criteria of the Literature

Inclusion Criteria: (1) Subject Relevance: the research content focuses on the thermal insulation performance, durability, or engineering applications of bio-based thermal insulation materials; (2) Document Type: research-oriented documents, including journal papers, theses and dissertations, conference papers, etc.; and (3) Language Type: Chinese or English documents.

Exclusion Criteria: (1) Duplicate Literature: the literature repeatedly included in different databases was excluded; (2) Non-research Literature: non-academic research documents such as news reports, product specifications, and patent abstracts were excluded; and (3) Low-relevance Literature: the literature that only mentions bio-based materials but does not involve the core content of thermal insulation performance and durability was excluded.

### 2.3. Literature Analysis Method

This study adopted a combination of qualitative integration and comparative analysis: (1) Qualitative Integration: the screened literature was subjected to content classification to organize the core information such as the classification, preparation processes, and performance indicators of bio-based thermal insulation materials. (2) Comparative Analysis: The thermal insulation performance and durability data of different materials were compared to analyze the influence rules of environmental factors on material properties. Meanwhile, the controversial research conclusions in this field were synthesized to explore the causes of discrepancies.

### 2.4. Count the Number of Literature by Material Category

After screening, a total of 103 valid literature were finally included in this study, with the statistics by material category as follows:

1. Natural biomass thermal insulation materials: 58 papers in total, covering straw bales, cork boards, hemp concrete, bamboo fiber materials, etc., focusing mainly on the native properties and basic modification research of the materials;
2. Bio-based composite thermal insulation materials: 45 papers in total, covering biomass–inorganic composites, biomass–polymer composites, and other types, focusing mainly on the performance optimization and engineering application research of the materials.

## 3. Bio-Based Materials

### 3.1. Classification of Bio-Based Insulation Materials

The bio-based thermal insulation materials defined in this study refer to building materials with heat insulation function, which are prepared from plant- or animal-derived biomass as the core raw material through physical processing, chemical modification, or composite molding, and mainly include three forms: (1) raw fiber type: such as loose straw fiber and hemp fiber, which are directly filled in building cavities; (2) molded board type: such as cork board and bamboo fiber board, which are formed by pressing or molding; and (3) composite modified type: such as straw–cement composite board and hemp fiber-PLA composite board, whose performance is improved by adding inorganic or organic binders.

The target building application scenarios for this category of materials include internal/external thermal insulation systems for building exterior walls, roof insulation layers, and infill layers for partition walls between households. The suitable climatic backgrounds are as follows: dry and cold regions (where the thermal insulation advantages are prioritized), mild and humid regions (requiring moisture-proof treatment), and coastal salt spray regions (requiring anti-corrosion modification).

#### 3.1.1. Natural Biomass Insulation Material

**Figure 1:**
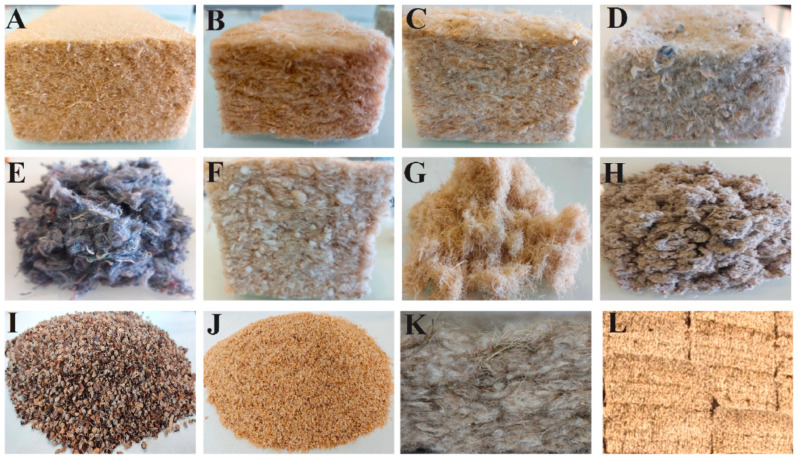
Partial biomass-based thermal insulation materials and products. (**A**) Densified wood board, (**B**) medium-density fiberboard (MDF), (**C**) hemp board, (**D**) cellulose-filled board, (**E**) loose-fill recycled textiles, (**F**) blended fiber (cotton/flax/hemp) board, (**G**) hemp fiber, (**H**) loose-fill cellulose insulation, (**I**) buckwheat hulls, (**J**) broomcorn millet hulls, (**K**) flax board, and (**L**) wheat straw bales [16].

Natural biomass thermal insulation materials are directly processed from a single type of plant or animal biomass without complicated composite modification. Typical examples include straw bales, cork boards, hemp concrete, and bamboo fiber thermal insulation materials. Straw bales made from wheat straw, rice straw, and other crop stalks are low-cost and widely available, and their natural pore structure endows them with inherent thermal insulation performance [15]. Cork boards are derived from the bark of cork oak trees, featuring a closed-cell structure with a porosity as high as 80–90%. They exhibit excellent thermal stability and a low thermal conductivity ranging from 0.035 to 0.040 W/(m·K), yet their production is restricted by regional resource distribution. Hemp concrete is a mixture of hemp core and lime-based binders, characterized by light weight (with a bulk density of 400–800 kg/m^3^) and air permeability, and has been extensively applied in low-energy-consumption buildings across Europe [22]. Bamboo fiber thermal insulation materials are prepared via processes such as bamboo crushing, degumming, and drying, which offer good mechanical properties and environmental friendliness, but their moisture absorption resistance needs to be further improved [23]. Figure 1 shows some biomass-based thermal insulation materials and their products [16].

#### 3.1.2. Bio-Based Composite Insulation Materials

**Figure 2:**
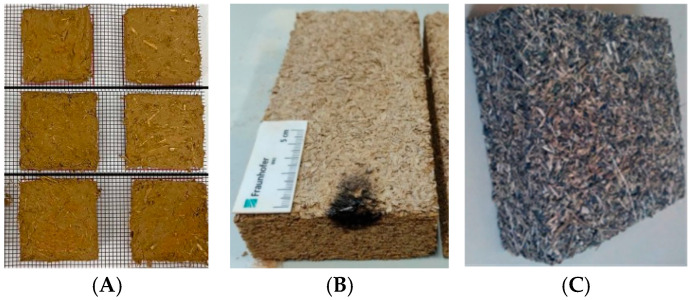
Partial bio-based composite insulation materials [26,27,28]. (**A**) Straw gypsum composite material [26]. (**B**) Rice straw clay composite material [27]. (**C**) Sunflower straw fiberboard [28].

Figure 2 shows some bio-based composite thermal insulation materials. Bio-based composite thermal insulation materials are formed by compounding biomass raw materials with binders, modifiers, or other functional materials, which are intended to make up for the defects of single natural biomass materials. According to the types of composite components, they can be classified into biomass–inorganic composite materials and biomass–polymer composite materials. Biomass–inorganic composite materials (e.g., straw–cement composite insulation boards and wood fiber–gypsum boards) combine the low thermal conductivity of biomass with the high strength and moisture resistance of inorganic materials, with a thermal conductivity generally ranging from 0.045 to 0.070 W/(m·K) [24]. Biomass–polymer composite materials (e.g., hemp fiber–polylactic acid (PLA) composite insulation materials and wood flour–polypropylene (PP) composite boards) adopt biodegradable polymers as binders. While retaining environmental friendliness, these materials enhance mechanical properties and durability, yet they are relatively high in cost [25].

### 3.2. Preparation Process of Bio-Based Insulation Materials

#### 3.2.1. Molding Process

Molding technology constitutes the core step in the preparation of bio-based thermal insulation materials, mainly including compression molding, extrusion molding, and casting molding. Compression molding is widely applied in the production of straw bales, wood fiber insulation boards, and similar products. It shapes materials by applying pressure at a specific temperature, and the density and thermal insulation performance of products can be adjusted by controlling the pressure (1–5 MPa) and temperature (80–150 °C) [29]. Extrusion molding is suitable for the continuous production of profiles such as bamboo fiber insulation strips. The material is melted and then extruded through a mold, with foaming agents added to form a porous structure, resulting in high production efficiency [30]. Casting molding is primarily used for materials like hemp concrete: the mixture of biomass raw materials and binders is poured into molds and cured at room or low temperature. Featuring a simple process, it is suitable for on-site construction [22].

#### 3.2.2. Modification Process

Modification technology is an important means to improve the performance of bio-based thermal insulation materials, including physical modification, chemical modification, and biological modification. Physical modification (e.g., carbonization and plasma treatment) alters the surface structure and pore characteristics of biomass materials. For instance, carbonization treatment (conducted at 200–500 °C) can reduce the moisture absorption rate of wood fibers by 30–50% and enhance their thermal stability [31]. Chemical modification (e.g., esterification, etherification, and silane modification) modifies the surface functional groups of biomass materials to improve their compatibility with binders and resistance to environmental factors. Silane modification can decrease the water absorption rate of straw fibers by 25–40% and boost the mechanical properties of composite materials [29]. Biological modification utilizes microorganisms or enzymes to treat biomass materials, which removes impurities and improves material performance. This method boasts excellent environmental friendliness yet imposes stringent requirements on reaction conditions [30].

## 4. Thermal Insulation Performance and Influencing Factors

### 4.1. Core Evaluation Indicators and Testing Methods

#### 4.1.1. Core Evaluation Indicators

The thermal insulation performance of bio-based thermal insulation materials is mainly evaluated by three core indicators: thermal conductivity (λ), thermal resistance (R), and thermal storage coefficient (S). Thermal conductivity is the most direct indicator reflecting thermal insulation capacity; the lower the value, the better the thermal insulation performance. High-quality bio-based thermal insulation materials typically have a thermal conductivity below 0.045 W/(m·K). Thermal resistance is the reciprocal of thermal conductivity per unit thickness (R = δ/λ, where δ denotes material thickness), which reflects the material’s ability to resist heat transfer. Thermal resistance of thermal insulation materials for external building walls is generally required to be ≥1.5 (m^2^·K)/W. The thermal storage coefficient reflects the material’s capacity to absorb and release heat, which affects indoor temperature stability. Bio-based materials generally have a moderate thermal storage coefficient (0.8–1.5 W/(m^2^·K)), which is conducive to regulating the indoor thermal environment.

#### 4.1.2. Testing Methods

Commonly used testing methods for thermal conductivity include the hot wire method (GB/T 10294-2008) [32], heat flow meter method (GB/T 10295-2008) [33], and guarded hot plate method (ISO 8302:1991) [34]. Different testing equipment and methods exert a significant influence on the test results of thermal conductivity, with a detailed analysis of the specific differences presented as follows:

1. Guarded Hot Plate Method: As an internationally recognized reference method, it features the highest testing accuracy (error < 2%). However, it requires complex equipment and involves a long testing cycle (8–12 h for a single sample), making it suitable for precise laboratory testing. Most of the basic data reported in the literature were obtained via this method [35].
2. Heat Flow Meter Method: It offers fast testing speed (1–2 h for a single sample) and enables on-site testing, yet it is susceptible to interference from ambient temperature and wind speed, with a testing error of approximately 5–8%, making it suitable for random sampling inspection of material properties at engineering sites.
3. Hot Wire Method: It is suitable for materials with low thermal conductivity and features high testing efficiency, yet it has strict requirements for sample homogeneity. When a sample contains pores or defects, the deviation of test results can reach more than 10%.

In addition, some studies have adopted the transient thermal analyzer for testing. This method is suitable for the rapid screening of small-sized samples, and the obtained data can be used as qualitative references but not as a basis for engineering design.

The testing of thermal resistance and thermal storage coefficient is usually derived from thermal conductivity test results through calculations incorporating material thickness and physical parameters, or directly measured by a thermal property tester [36].

#### 4.1.3. Correlation Table of Thermal Conductivity and Testing Conditions

To enable a rational comparison of research data from different studies, this study collated the core data of the included literature and constructed Table 1 to clarify the correlation between thermal conductivity, testing methods, and boundary conditions.

### 4.2. Key Influencing Factors of Insulation Performance

#### 4.2.1. Internal Factors of Materials

The type and proportion of biomass raw materials and binders directly affect the thermal insulation performance. For example, in hemp concrete, increasing the content of hemp core can reduce the thermal conductivity, yet excessive addition will lead to a decline in mechanical properties. In biomass–inorganic composite materials, an increase in the content of inorganic binders (e.g., cement and gypsum) will cause the thermal conductivity to rise. Porosity, pore size, and pore type are the core factors affecting thermal conductivity, with closed pores being more conducive to thermal insulation than open pores. The thermal insulation performance of materials reaches the optimum when the porosity ranges from 70% to 90% and the average pore size is 1–10 μm; excessively large pore sizes (>50 μm) will induce air convection inside the materials, thereby increasing the thermal conductivity [37]. Bio-based materials have an optimal density range for thermal insulation performance. When the density is too low (<300 kg/m^3^), the pore structure is loose and air convection is intense. When the density is too high (>800 kg/m^3^), the heat transfer paths multiply. The optimal density range for most bio-based thermal insulation materials is 300–600 kg/m^3^ [38].

#### 4.2.2. External Environmental Factors

**Figure 3:**
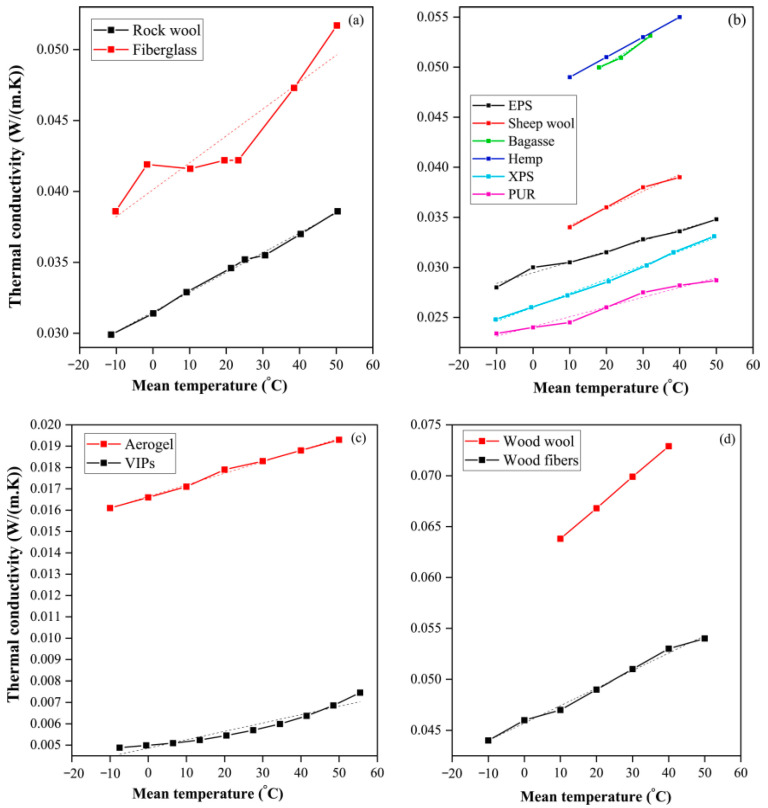
The influence of average temperature on the thermal conductivity of various building insulation materials: (**a**) inorganic materials; (**b**) organic materials; (**c**) advanced materials; and (**d**) composite materials [40].

Within the temperature range of 0–60 °C, the thermal conductivity of bio-based materials increases with rising temperature. For instance, the thermal conductivity of straw bales can increase by 10–15% when the temperature rises from 20 °C to 50 °C [39]. This is because the thermal motion of molecules inside the materials and air convection are enhanced at high temperatures. Figure 3 illustrates the effect of average temperature on the thermal conductivity of various building insulation materials [40]. A comparison shows that the thermal conductivity of bio-based materials (e.g., hemp and bagasse) is less sensitive to temperature changes than that of inorganic materials (e.g., rock wool), but slightly more sensitive than that of traditional organic materials (e.g., EPS). This indicates that in high-temperature environments, the thermal insulation performance of bio-based materials degrades to a lesser extent than that of rock wool, making them more suitable for the external wall insulation of buildings in hot regions.

**Figure 4:**
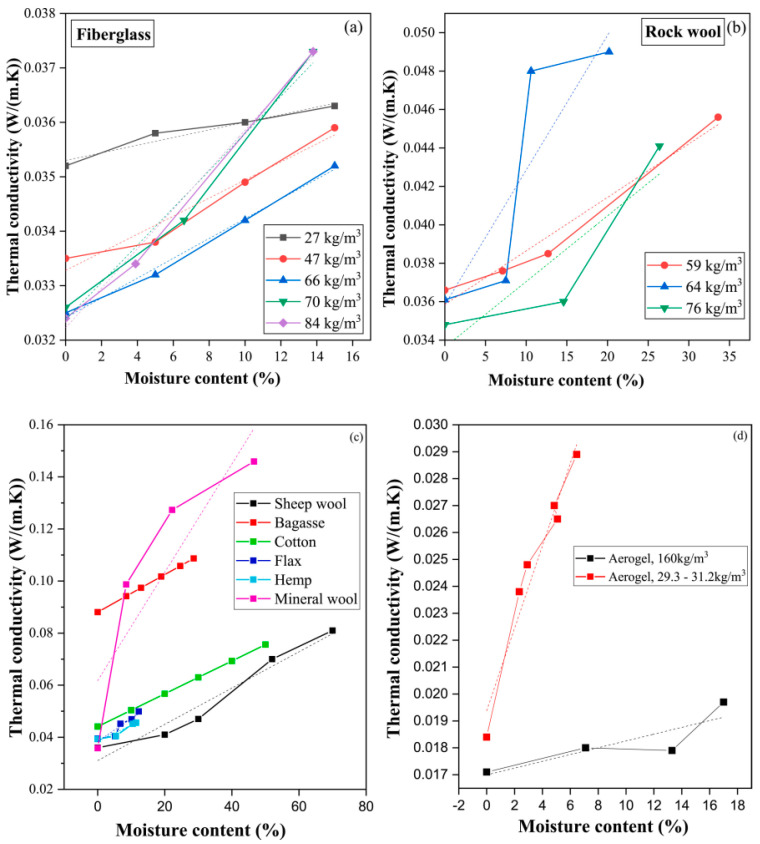
The influence of moisture content of different building insulation materials on thermal conductivity: (**a**) glass fiber; (**b**) rock wool; (**c**) natural materials; and (**d**) aerogel [40].

Moisture absorption is a key factor leading to the deterioration of thermal insulation performance. When the moisture content of the material increases from 0% to 20%, its thermal conductivity can rise by 30–60% [41]. Water molecules fill the pores and form heat transfer channels, which significantly enhance the heat transfer efficiency. Figure 4 depicts the effect of moisture content on the thermal conductivity of different building insulation materials [40]. The key conclusions are as follows: the increase in thermal conductivity of bio-based materials with the rise in moisture content is significantly higher than that of traditional thermal insulation materials. When the moisture content exceeds 20%, the increase in their thermal conductivity can reach 30–60%. This explains the core reason for the poor thermal insulation performance of bio-based materials in humid regions and also points out the direction for material modification—prioritizing the improvement of moisture resistance.

The higher the velocity of air movement on the surface, the higher the heat transfer rate, and thus the higher the surface coefficient [42]. The convective heat transfer coefficient of the external surface is a function of windward velocity, which ranged from 1 to 10 m/s in one study. Based on this relationship, the higher the velocity of air movement on the surface, the higher the rate of heat transfer through the building envelope. Therefore, a higher heat transfer coefficient results in a smaller temperature difference between the outdoor and indoor environments, which in turn impairs the thermal insulation performance of insulation materials.

### 4.3. Case Studies on in Situ Measurement

Laboratory test data can only reflect the performance of materials under controlled conditions, whereas in situ measurement can more truly represent their service performance in actual buildings. Latif et al. [43] selected wood-framed wall panels with and without vapor barriers, filled them with wood-hemp insulation materials, and conducted a 12-month in situ monitoring. The results showed that the moisture content of hemp materials in the wall panels with vapor barriers remained stable at 8–10%, with the thermal conductivity maintained at 0.040–0.042 W/(m·K); in the wall panels without vapor barriers, the moisture content of the materials fluctuated by 15–20% with seasons, the thermal conductivity rose to a maximum of 0.058 W/(m·K), and the thermal insulation performance degraded by approximately 30%. This study confirmed that the installation of vapor barriers can effectively mitigate the performance degradation of bio-based materials caused by moisture absorption. In another in situ study on experimental walls of hemp–lime composite materials, Latif et al. [44] found that such materials exhibit good moisture regulation potential and can reduce the fluctuation range of indoor relative humidity by 15–20%. However, during the plum rain season, the moisture absorption of the materials leads to a decrease in thermal resistance by about 12%, necessitating the matching design of breathable structures.

### 4.4. Performance Comparison with Traditional Thermal Insulation Materials

Table 2 compares the core properties of common bio-based and traditional thermal insulation materials, with the comparison benchmarks being the same test environment (temperature: 20 °C, relative humidity: 50%) and the same thickness (50 mm). It can be observed that the thermal conductivity of natural biomass materials (e.g., cork boards and hemp concrete) is comparable to that of traditional materials such as expanded polystyrene (EPS) and mineral wool, with some even demonstrating superior performance. Owing to the incorporation of inorganic binders, bio-based composite materials have relatively higher thermal conductivity, but they exhibit distinct advantages in environmental performance, including renewability, biodegradability, and carbon footprint [45]. However, the thermal insulation performance of bio-based materials is more sensitive to humidity, which constitutes a key issue to be resolved in engineering applications.

The comparison results under comparable conditions show that the thermal insulation performance of natural bio-based materials (cork board and hemp concrete) is basically on par with that of traditional materials, with some indicators even superior; however, the moisture absorption rate of bio-based materials is significantly higher than that of traditional materials, which constitutes the core shortcoming of their engineering application. For bio-based composite materials, the incorporation of inorganic binders reduces the moisture absorption rate yet leads to a slight increase in thermal conductivity, reflecting a trade-off among various performance properties.

### 4.5. Interaction and Trade-Off Framework for Thermal Insulation Performance–Moisture Sensitivity–Durability–Mechanical Properties

Based on the above research, to systematically integrate the coupling relationships among various properties, this study constructed a multi-property interaction and trade-off analysis framework for bio-based thermal insulation materials:

(1) Negative correlation between thermal insulation performance and moisture sensitivity: the higher the material porosity, the better the initial thermal insulation performance; however, more moisture absorption channels will result in a greater increase in thermal conductivity after moisture absorption and a more significant attenuation of thermal insulation performance.
(2) Coupling relationship between moisture sensitivity and durability: moisture absorption can induce fiber swelling and interface debonding, which in turn accelerates material cracking and biological erosion under temperature–humidity cycles, leading to a significant reduction in durability.
(3) Trade-off relationship between thermal insulation performance and mechanical properties: improving thermal insulation performance requires an increase in porosity, yet high porosity will cause a decrease in the mechanical strength of materials and raise the risk of damage during construction and service.
(4) Reverse effect of durability attenuation on thermal insulation performance: Structural damage to materials caused by corrosion and biological erosion during service will introduce additional heat transfer paths, further deteriorating thermal insulation performance.

This framework can provide guidance for material formulation design, i.e., it is necessary to seek an optimal balance among thermal insulation performance, mechanical properties, and durability, rather than pursuing the optimization of a single property alone.

## 5. Durability and Attenuation Mechanism

### 5.1. Evaluation Dimensions and Testing Standards for Durability

The durability of bio-based thermal insulation materials refers to their ability to maintain stable performance under the action of various environmental factors during service. The core evaluation dimensions include temperature–humidity stability, corrosion resistance, biological erosion resistance, and mechanical durability. Relevant testing standards have been formulated in various countries, such as China’s Materials for External Thermal Insulation Composite Systems Based on Molded Polystyrene Boards (GB/T 29906-2013) [46], ISO’s Plastics—Evaluation of the Action of Micro-organisms (ISO 846:2019) [47], and ASTM’s Standard Test Method for Evaluating Odor Emission from Thermal Insulation Materials (ASTM C1304-18) [48]. These standards specify the testing conditions (e.g., temperature–humidity cycling parameters, corrosive medium concentration, and testing duration) and evaluation indicators (e.g., performance retention rate and mass loss rate), so as to ensure the comparability of test results. In practical application, the durability of bio-based thermal insulation materials tends to degrade under the combined influence of multiple factors.

The core differences between laboratory research and practical engineering applications can be summarized into two points: first, the complexity of influencing factors—single variables are mostly controlled in the laboratory, while multiple factors are coupled in practical engineering; second, the discrepancy in time scales—the excessively high time compression ratio of laboratory accelerated tests makes it difficult to simulate the slow degradation process of materials during long-term service. Therefore, the material service life derived from laboratory tests (15–20 years) needs to be revised to the actual engineering service life (10–15 years), and this revised value can provide a more accurate reference for engineering design.

### 5.2. Key Durability Performance and Attenuation Mechanism

#### 5.2.1. Temperature and Humidity Stability

**Figure 5:**
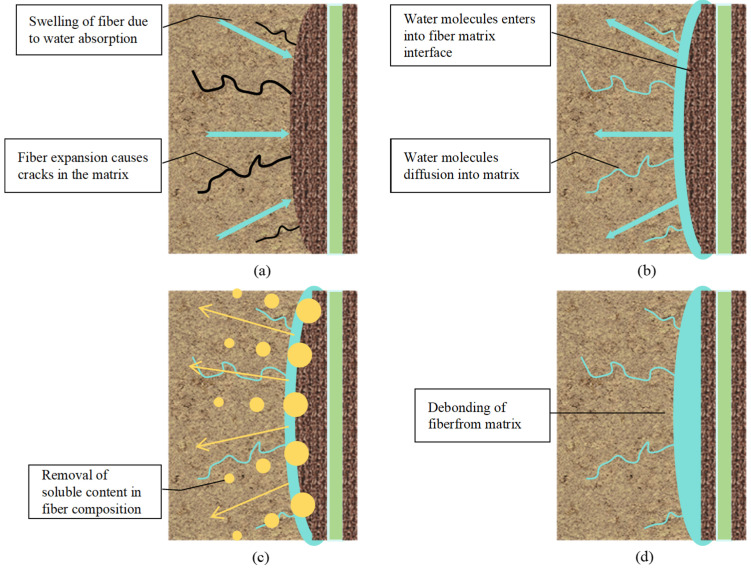
The main mechanism of water absorption in bio-composite materials. (**a**) Process 1; (**b**) Process 2; (**c**) Process 3; (**d**) Process 4.

Temperature–humidity cycling is one of the primary factors causing performance degradation of bio-based thermal insulation materials. Under the action of repeated temperature variations (−20 °C to 60 °C) and humidity fluctuations (30% RH to 90% RH), materials undergo thermal expansion and contraction, as well as moisture absorption–desorption cycles, which trigger cracking, deformation, and even structural damage [40,49]. Figure 5 illustrates the main mechanisms of moisture absorption in bio-composites. The degradation mechanisms mainly involve two aspects. First, the difference in thermal expansion coefficients between biomass components and binders leads to interface separation. Second, the swelling and shrinkage of biomass fibers caused by moisture absorption–desorption result in the accumulation of internal stress [50,51]. After 50–100 cycles of temperature and humidity, the thermal conductivity of most bio-based materials increases by 15–30%, while their mechanical strength decreases by 20–40% [52,53,54].

#### 5.2.2. Corrosion Resistance

Corrosion resistance mainly refers to the material’s ability to resist acid, alkali, and salt media in the environment. In industrial zones or coastal environments, bio-based materials are susceptible to erosion by acid rain (pH = 3–5) or salt spray (sodium chloride concentration 3–5%), which leads to component degradation and performance attenuation [55]. The degradation mechanisms are as follows: acidic media can decompose cellulose and hemicellulose in biomass materials, reducing structural integrity; alkaline media can react with lignin, causing fiber swelling and softening; and salt ions can penetrate into the interior of materials to form crystalline precipitates, resulting in pore blockage or structural cracking. Bio-based composite materials exhibit better corrosion resistance than natural biomass materials. For example, straw–cement composite boards can retain 70–80% of their original performance after 30 days of acid–alkali immersion, while pure straw bales only retain 40–50% [49].

#### 5.2.3. Resistance to Biological Erosion

**Figure 6:**
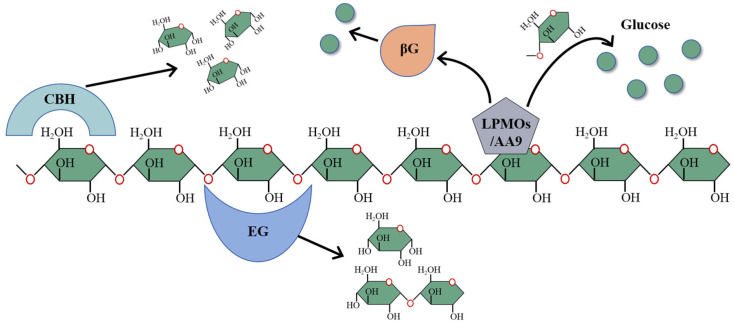
Mechanism of cellulose biodegradation.

Biological erosion resistance is a unique challenge faced by bio-based materials. Biomass materials with high cellulose and hemicellulose contents are prone to infestation by microorganisms such as fungi, bacteria, and insects (e.g., termites and wood borers), which leads to material decay and performance loss [56]. The erosion mechanism lies in the fact that microorganisms secrete enzymes to decompose cellulose and hemicellulose into nutrients required for their own growth, thus damaging the material structure [57,58,59]. Figure 6 shows the mechanism of cellulose biodegradation. Natural biomass materials exhibit poor biological durability; for example, unmodified wood fiber insulation boards show obvious decay after 6 months of exposure to humid environments, with a mass loss rate of 15–25% [60]. Chemical modification (e.g., adding preservatives and fungicides) or physical modification (e.g., carbonization) can improve the biological erosion resistance.

#### 5.2.4. Mechanical Durability

Mechanical durability refers to the ability of materials to resist mechanical wear, impact, and pressure during transportation, construction, and service. Bio-based materials generally exhibit low mechanical strength and are prone to damage under external forces, which leads to the deterioration of thermal insulation performance [61]. The degradation mechanism lies in the fact that mechanical actions cause the fracture of biomass fibers, damage to pore structures, and separation of composite interfaces. The mechanical durability of bio-based composite materials is significantly superior to that of natural biomass materials. For instance, the impact strength of hemp fiber–polylactic acid (PLA) composites is 3–5 times that of pure hemp concrete, with a mass loss rate of only 2–5% after 1000 wear cycles [61].

### 5.3. Durability Evolution Matrix for Bio-Based Thermal Insulation Materials

To conduct a structured analysis of the long-term retention laws of thermal and structural properties of bio-based thermal insulation materials, this study collated test data with different exposure durations from the existing literature and constructed Table 3: Durability Evolution Matrix, which clearly presents the quantitative characteristics of performance degradation.

As can be seen from the matrix table, the durability performance of materials is closely related to exposure conditions and material types: natural biomass materials exhibit significant performance degradation under the combined action of multiple factors, while composite modified materials have a higher performance retention rate; biological erosion has a greater impact on the performance of natural materials than temperature–humidity cycles, whereas salt spray erosion has a relatively mild effect on composite materials. This matrix can provide a quantitative reference for the prediction of material service life in engineering design.

## 6. Engineering Application Challenges and Optimization Paths

### 6.1. Key Challenges in Engineering Applications

#### 6.1.1. Insufficient Stability of Insulation Performance

The thermal insulation performance of bio-based thermal insulation materials is highly sensitive to environmental humidity, which constitutes the biggest bottleneck restricting their engineering applications. In humid areas (relative humidity > 70%), the moisture absorption rate of such materials can reach 15–25%, leading to a significant increase in thermal conductivity that fails to meet the requirements of building energy-saving design. In addition, under the combined action of long-term temperature–humidity cycling and biological erosion, the thermal insulation performance of the materials will gradually degrade, with a service life of generally 10–15 years, which is shorter than that of traditional materials (20–30 years) [62,63].

#### 6.1.2. Poor Construction Adaptability

The poor construction compatibility of bio-based thermal insulation materials is mainly reflected in three aspects: first, their low mechanical strength makes them prone to fracture and deformation during cutting, transportation, and installation, which impairs construction quality [64]; second, their high water absorption rate requires moisture-proof measures to be taken during construction, thus increasing construction difficulty and cost; and third, their poor compatibility with traditional building materials (e.g., concrete and mortar) leads to low interface bonding strength, which is likely to cause problems such as hollowing and cracking [65,66,67].

#### 6.1.3. High Cost and Missing Standards

The cost of bio-based thermal insulation materials is usually 1.5–3 times that of traditional materials such as EPS and mineral wool, with the high cost mainly stemming from the prices of raw materials (e.g., cork and hemp) and the complexity of modification processes [68]. In addition, the lack of unified industry standards and technical specifications is another important factor restricting their engineering applications. At present, the performance evaluation indicators, testing methods, and construction technical requirements for bio-based thermal insulation materials have not yet been standardized, resulting in inconsistent product quality and great difficulty in engineering quality control.

### 6.2. Optimizing the Path

#### 6.2.1. Performance Improvement Strategy

Modification of materials can be achieved by combining physical methods with chemical approaches to improve their moisture resistance. For example, first subjecting biomass fibers to carbonization treatment to reduce hydrophilic groups, followed by coating a layer of hydrophobic agent (e.g., organosilicon and paraffin wax) on the surface to form a hydrophobic film, can reduce the moisture absorption rate of the materials to below 8%. The comprehensive optimization of thermal insulation and durability performance can be realized through the development of multi-component composite systems [69,70,71]. A typical example is the biomass fiber–inorganic filler–polymer composite material. Inorganic fillers (e.g., nano-silica and hollow glass microspheres) can improve thermal insulation performance and corrosion resistance, while polymers such as PLA and starch-based binders can enhance mechanical properties and biological durability.

Through genetic engineering or selective breeding, research can be conducted on customizing source plants (flax, jute, and bamboo) to enhance their traits (e.g., higher cellulose content and specific fiber morphology), thereby optimizing the thermal insulation performance [72].

#### 6.2.2. Improvement of Construction Adaptability

Explore the application of 3D printing technology in the processing of bio-based thermal insulation materials, investigate its impacts on the microstructure and anisotropic properties, as well as its potential for fabricating complex geometric shapes, and address the challenges associated with material formulation and large-scale production [73]. Improve the interface bonding performance: surface modification of bio-based materials (e.g., plasma treatment and silane coupling agent treatment [74,75]) can enhance their compatibility with traditional building materials; alternatively, develop binders dedicated to bio-based materials to increase the interface bonding strength [17,76,77]. Formulation of dedicated construction specifications: formulate targeted construction technical specifications according to the characteristics of bio-based materials, including moisture-proof measures, installation methods, and quality inspection standards during construction, so as to ensure construction quality.

#### 6.2.3. Cost Control and Standardization

Continue to carry out sustainable production and improve energy-saving and resource-efficient production methods. Emphasize rigorous life cycle assessment to identify low-impact processes and explore greener binders/treatment methods [73,78]. Optimize and improve production processes and develop efficient and low-cost production technologies. Examples include continuous extrusion molding technology and microwave drying technology, which enhance production efficiency and reduce energy consumption. Establishment of Unified Standards: Accelerate the formulation of national and industrial standards for bio-based thermal insulation materials, including product performance indicators, testing methods, construction technical specifications, and quality evaluation systems, so as to regulate market order and promote large-scale application [79,80].

### 6.3. Material Selection and Design Guidelines for Engineering Practice

To translate the research findings into specific guidelines for engineering applications, this study refines the following principles for material selection and design:

1. Principles of Material Selection:
  (1) Arid regions should prioritize the use of natural bio-based materials (e.g., cork boards and straw bales) to fully leverage their advantages in thermal insulation performance;
  (2) Humid/coastal regions require the adoption of hydrophobically modified bio-based composite materials (e.g., straw–cement composite boards) to mitigate the impact of moisture absorption on material performance;
  (3) Scenarios with high mechanical performance requirements (e.g., external wall hanging boards) should employ biomass–inorganic composite systems to balance mechanical strength and thermal insulation performance.
2. Key Points of Engineering Design:
  (1) Exterior wall thermal insulation systems shall be equipped with a moisture barrier to block the intrusion of external moisture, and a breathable layer shall be installed simultaneously to discharge internal water vapor of the materials;
  (2) The moisture content of materials shall be controlled below 10% during construction to avoid cracking caused by wet expansion and dry shrinkage;
  (3) Material thickness shall be adjusted for different climatic zones and appropriately increased in cold regions to meet the thermal resistance requirements.

## 7. Conclusions and Future Prospects

### 7.1. Conclusions

Based on a comprehensive analysis of the relevant literature published in the past decade, this review systematically summarizes the research progress of bio-based thermal insulation materials in terms of thermal insulation performance, durability, and engineering applications. The key conclusions are as follows:

(1) Bio-based thermal insulation materials are classified into two categories: natural biomass materials and bio-based composite materials. The former exhibits excellent thermal insulation performance (with a thermal conductivity of 0.034–0.055 W/(m·K)) but poor durability; the latter balances thermal insulation and durability through component compounding yet has a slightly higher thermal conductivity (0.045–0.070 W/(m·K)).
(2) The thermal insulation performance of bio-based materials is jointly affected by internal factors (component ratio, pore structure, and density) and external factors (temperature, humidity, and atmospheric pressure). Among these, pore structure (with a porosity of 70–90% and a closed-cell ratio of >80%) and moisture content are the most critical influencing factors.
(3) The durability of bio-based materials is restricted by temperature–humidity stability, corrosion resistance, biological erosion resistance, and mechanical durability. Temperature–humidity cycling and biological erosion are the primary causes of performance degradation, and composite modification is an effective approach to enhance durability.
(4) The engineering application of bio-based thermal insulation materials faces three key challenges: insufficient stability of thermal insulation performance, poor construction compatibility, high cost, and lack of uniform standards. Multi-scale modification, composite optimization, and standardized construction are the core solutions to address these problems.

Through the aforementioned analyses, this study has achieved the four preset research objectives: (1) systematically integrating the core thermal insulation indicators and their influencing factors; (2) revealing the degradation laws and mechanisms of material durability in complex environments; (3) clarifying the core focuses and causes of discrepancies in the debates on environmental performance; and (4) proposing targeted approaches for performance optimization and engineering application.

### 7.2. Future Outlook

Based on the current research status and existing problems, the future research directions of bio-based thermal insulation materials are proposed as follows:

(1) Development of high-performance composite systems: Focus on the research and development of biomass–nanocomposites and biomass–intelligent composites. Nanomaterials (e.g., graphene and carbon nanotubes) are utilized to enhance thermal insulation performance and durability, while intelligent materials (e.g., shape memory polymers) are integrated to realize self-regulation of thermal insulation performance, thus meeting the requirements of complex environmental conditions.
(2) In-depth research on long-term durability mechanisms: conduct long-term (>20 years) tracking tests on bio-based materials in different climatic zones (humid, cold, coastal areas, etc.), clarify the long-term performance degradation rules and mechanisms, and establish durability prediction models to provide a scientific basis for service life design.
(3) Innovation of low-cost green preparation technologies: develop low-energy-consumption production technologies such as formaldehyde-free bio-based binders and solar-assisted drying, expand the use of industrial and agricultural forestry wastes as raw material sources, and achieve the unification of environmental friendliness and low cost.
(4) Improvement of standardization and industrialization levels: Accelerate the improvement of a standard system covering raw materials, production, performance testing, construction, and acceptance. Promote the industrialization of bio-based thermal insulation materials through pilot projects and demonstration programs, so as to realize their large-scale application in the construction industry.

## Figures and Tables

**Table 1:** Correlation Table of Thermal Conductivity and Testing Conditions for Bio-based Thermal Insulation Materials.

Material·Type	Thermal Conductivity λ (W/(m·K))	Testing Method	Boundary Conditions (Temperature/Humidity)	Density (kg/m^3^)	Humidity Adjustment Conditions
Cork·board	0.034–0.040	Guarded Hot Plate Method	20 °C/50%RH	150–250	Absolutely dry state
Hemp concrete	0.038–0.045	Guarded Hot Plate Method	20 °C/50%RH	400–800	Air-dried naturally
Straw cement composite board	0.045–0.060	Heat Flow Meter Method	25 °C/60%RH	500–700	Moisture content 10%
Bamboo fiberboard	0.042–0.048	Hot Wire Method	20 °C/45%RH	300–400	Absolutely dry state

**Table 2:** Comparison of insulation performance between bio-based and traditional insulation materials.

Material Type	Thermal Conductivity λ (W/(m·K))	Density (kg/m^3^)	Thermal Resistance Value R (m^2^·K)/W (δ = 50 mm)	Moisture Absorption Rate (%)
Cork board (bio-based)	0.034–0.040	150–250	1.25–1.47	8–12
Hemp concrete (bio-based)	0.038–0.045	400–800	1.11–1.32	15–20
Straw cement composite board (bio-based)	0.045–0.060	500–700	0.83–1.11	10–15
Polystyrene foam (EPS, traditional)	0.039–0.042	20–40	1.19–1.28	2–4
Mineral wool (traditional)	0.036–0.040	60–100	1.25–1.39	4–8

**Table 3:** Durability Evolution Matrix Table for Bio-based Thermal Insulation Materials.

Material Type	Exposure Conditions	Exposure Duration	Change in Thermal Performance (Increase in Thermal Conductivity)	Change in Structural Performance (Reduction in Compressive Strength)
Pure straw bales	Natural temperature and humidity cycle	1 year	18%	25%
Pure straw bales	Natural temperature and humidity cycles + biological erosion	2 years	35%	42%
Straw-cement composite board	Natural temperature and humidity cycle + salt spray erosion	2 years	12%	15%
Hemp concrete	Accelerate temperature and humidity cycling (100 times)	-	22%	30%
Cork board	Natural exposure to sunlight + rain	3 years	10%	8%

## Data Availability

No new data were created or analyzed in this study. Data sharing is not applicable to this article.
